# Cross-Model Explainability Consistency in Hepatitis C Stage Classification: A SHAP, LIME, and Counterfactual Analysis Across Five Machine Learning Architectures

**DOI:** 10.3390/diagnostics16142197

**Published:** 2026-07-14

**Authors:** Khalid Alalawi

**Affiliations:** College of Computer Science and Engineering (CCSE), Taibah University, Medina 42353, Saudi Arabia; kalwai@taibahu.edu.sa

**Keywords:** hepatitis C, explainable AI, SHAP, LIME, counterfactual explanations, cross-model agreement, machine learning diagnostics, clinical decision support, liver disease staging

## Abstract

**Background**: Hepatitis C virus (HCV) affects approximately 50 million people worldwide and progresses silently through distinct hepatic stages, yet most machine learning staging approaches offer no clinician-facing explanation and rarely evaluate whether explanations hold across architectures. **Methods**: We trained five models—Logistic Regression, Random Forest, XGBoost, LightGBM, and SVM—on the UCI HCV dataset (615 patients, four classes after merging the seven-instance suspect blood-donor group into a single Donor/Control class) and applied SHAP, LIME, and DiCE counterfactuals. A cross-model Spearman agreement analysis quantified feature ranking consistency, with a leakage-controlled pipeline and five-fold stratified cross-validation applied. **Results**: In the main 5-fold cross-validation, LightGBM achieved the highest macro-F1 (0.684 ± 0.031). Under repeated-seed cross-validation, XGBoost and LightGBM gave closely matched values (0.648 ± 0.023 and 0.645 ± 0.026), indicating comparable robustness among the boosted-tree models, both marginally ahead of the remaining three, with Random Forest close behind. Logistic Regression reached the highest macro-F1 on the single held-out split (0.790), but its cross-validated score was markedly lower (0.598 ± 0.063), underlining how unstable single-split estimates are in a small, imbalanced cohort. SHAP consistently identified AST, GGT, CHE, and ALP across the tree-based models, with CHE emerging as the leading Cirrhosis-stage marker in the boosted models. Cross-model Spearman correlations reached 0.923–0.958 among tree-based models; SHAP-LIME overlap ranged from 1/5 to 4/5. **Conclusions**: The framework identifies stage-specific biochemical importance patterns consistent with known HCV disease progression. The convergent finding on CHE for Cirrhosis, supported by SHAP in the tree-based models and appearing among the top LIME features in three of the five model-specific Cirrhosis explanations, supports CHE as a candidate marker worth evaluating in advanced disease.

## 1. Introduction

Hepatitis C is still a major global health burden. Treatment has improved dramatically, and DAA therapy now clears the virus in around 95% of treated patients [1], yet most infected people worldwide are never diagnosed or treated. Left untreated, chronic HCV infection moves silently through a series of distinct hepatic stages—from initial Hepatitis and Fibrosis through to end-stage Cirrhosis, in which the liver progressively loses its synthetic capacity and the risk of hepatocellular carcinoma rises substantially. Even after achieving virological cure, cirrhotic patients need ongoing HCC surveillance and remain at substantially higher risk of end-stage liver disease complications [1].

The central challenge is that this progression is largely asymptomatic until advanced stages, and liver biopsy—the only investigation that can estimate the severity of tissue damage and the historical gold standard for HCV grading and staging [2]—is increasingly supplemented by noninvasive biochemical approaches. Routine biochemical markers obtainable from standard blood panels carry information about hepatic function: ALT and AST reflect hepatocellular injury and normalise rapidly following successful DAA therapy [3,4]; GGT may indicate hepatobiliary or cholestatic pathology [5]; albumin reflects hepatic synthetic capacity and declines progressively with disease severity [5]; cholinesterase similarly falls as hepatocyte mass is lost [6,7,8]; and bilirubin reflects hepatic conjugation and biliary excretion capacity [5]. These markers shift progressively across the HCV stage spectrum, from Donor/Control to Cirrhosis. Machine learning models can exploit complex non-linear interactions among these markers to support more flexible and potentially more accurate staging approaches than traditional rule-based scoring systems [9,10,11,12]. However, a fundamental barrier to clinical adoption is that ML models typically produce predictions with no explanation that a clinician can examine or question [13]. A hepatologist who cannot understand why a model predicts Cirrhosis cannot safely integrate that prediction into treatment planning, drug dosing, or surveillance decisions [13]. While Rudin [13] advocates for inherently interpretable models as the theoretical ideal, post hoc frameworks such as SHAP [14] and LIME [15] are the most practical way to open up high-performing ensemble models to clinical scrutiny when the data is tabular. An equally pressing but less studied question concerns whether XAI explanations hold up across different model architectures. Strong cross-model agreement would give clinicians much more reason to trust that a feature matters, rather than suspecting it is an artefact of one particular algorithm [16,17]. The prior HCV works reviewed here [9,10,11,12] rely on single train–test splits, which we demonstrate can produce misleading minority class estimates when disease classes contain only a handful of instances.

### 1.1. Global Burden and Clinical Context

HCV infection affects approximately 50 million people globally, with an estimated 1.0 million new HCV infections recorded in 2022 [1]. Combined viral Hepatitis (B and C) accounts for 1.3 million deaths per year—a mortality burden comparable to tuberculosis—with HCV responsible for approximately 240,000 deaths (~17%) [1]. Direct-acting antiviral (DAA) therapies now achieve sustained virological response (SVR) rates of approximately 95% [18], fundamentally changing what is possible for most patients. Nevertheless, HCV remains a leading cause of preventable death: most infections go undetected until advanced stages, by which point the opportunity for cure is often narrower [1]. This challenge is sharpest in the Eastern Mediterranean Region, which carries the highest HCV burden of any WHO region—approximately 12 million chronically infected individuals—where prevalence among key populations such as people who inject drugs reaches 31%, against just 2% in apparently healthy individuals [19]. The course of HCV infection is well understood. Approximately 55–85% of acutely infected individuals fail to clear the virus spontaneously and develop chronic infection [20]. Of those chronically infected, 15–30% progress to Cirrhosis over 20 years [20], and cirrhotic patients face a substantially elevated annual risk of hepatocellular carcinoma (HCC) depending on clinical and virological factors [21]. HCV-related Cirrhosis and HCC collectively account for the majority of HCV-attributable mortality [1]. Accurate biochemical staging of disease progression is therefore critical for guiding antiviral treatment timing and selection. In clinical practice, staging relies heavily on invasive liver biopsy—the histological gold standard—but biopsy carries procedural risk and is subject to sampling error and sampling variability [22]. Serum biomarkers offer a safer, more scalable alternative [22]. Those available in this dataset—AST, ALT, ALB, BIL, CHE, ALP, and GGT—each reflect a different facet of hepatic function [23]. However, their individual diagnostic accuracy is limited [22], and their combined interpretation across four disease stages (Donor/Control, Hepatitis, Fibrosis, and Cirrhosis) poses a demanding four-class classification challenge, one that calls for methods capable of learning complex, non-linear biomarker interactions.

### 1.2. Machine Learning for HCV Staging

Machine learning is now widely applied across the liver-disease pipeline, and recent reviews of AI applied to omics and clinical data in hepatology stress both its diagnostic promise and the need for interpretable, well-validated models before clinical adoption [24]. Within HCV staging specifically, Alizargar et al. (2023) [9] benchmarked six algorithms including SVM and XGBoost on both the UCI HCV dataset and the NHANES dataset under a binary HCV-positive versus -negative formulation, achieving accuracy of 0.95 and AUC of 0.984. Both SVM and XGBoost tied as the best-performing models; feature importance analysis placed AST first and ALP second on the UCI dataset [9]. Butt et al. (2021) [10] proposed an ANN-based system (IHSDS) on an Egyptian HCV cohort (HCV-Egy dataset, 1385 samples, 29 features, 4 histological stages) achieving 94.44% validation precision—though using a proprietary metric on a structurally different dataset, limiting direct comparability. Jangiti et al. (2023) [11] applied five classifiers to a three-class HCV severity formulation on an oversampled version of the UCI dataset. Samreen (2024) [25] proposed a stacking ensemble achieving F1 = 0.95 on a small Kanazawa University dataset (123 records), employing repeated stratified K-fold CV—the only prior HCV work to do so systematically. Hossain et al. (2025) [12] evaluated seven ML algorithms including LightGBM with SMOTE on the UCI HCV dataset using an 80/20 hold-out split. Das et al. (2025) [26] applied SHAP-based explainable ML to Hepatitis prediction, demonstrating that SVM with hyperparameter tuning achieves 99.25% accuracy on a Hepatitis classification dataset. Alizargar et al. [9] used a binary formulation that collapses Hepatitis, Fibrosis, and Cirrhosis into a single positive class, making stage-specific F1 scores undefined, while Butt et al. [10], Jangiti et al. [11], and Hossain et al. [12] each rely on a single hold-out split. Among the works reviewed here, none provide cross-validated per-class F1 scores for individual minority disease stages, a gap this study addresses directly. Recent studies have applied a range of validation and feature-selection strategies to Hepatitis classification. Khatun et al. [27] compared a Logistic Regression baseline against six machine learning classifiers on the UCI Hepatitis dataset with Boruta feature selection, evaluated under repeated 10-fold cross-validation, but on a binary Die/Live outcome rather than multi-class staging. Sunori et al. [28] applied PCA-based dimensionality reduction to the same 615-record HCV biomarker panel used here before multi-class staging, though under a single stratified 80/20 split. Neither report cross-validated F1 for the individual minority disease stages, and neither carry out a formal cross-model explanation-agreement analysis. In a much larger clinical setting, Lu et al. [29] combined XGBoost with SHAP on a nationwide HCV registry to predict direct-acting antiviral failure and reported bilirubin among the leading predictors, with AST among the contributing features; both markers also appear in the present analysis. On the explanation side, recent counterfactual research such as DiCE-Extended [30] has focused on improving the robustness and feasibility of generated counterfactuals, complementing the immutability constraints of the original DiCE framework [31] that we adopt here for demographic features. The reliability of the explanations themselves is an active concern: Salih et al. [32] show that SHAP and LIME can disagree and that feature collinearity distorts both, which is precisely why the cross-method and cross-model agreement analysis reported here is worth carrying out rather than relying on a single explainer.

### 1.3. Explainable AI: SHAP

SHAP, introduced by Lundberg and Lee (2017) [14] at NeurIPS, unified six feature attribution methods within a cooperative game theory framework, providing consistency and local accuracy guarantees that earlier methods lacked. TreeExplainer computes exact Shapley values for tree-based models using the tree structure [14,33], while KernelExplainer provides a model-agnostic approximation. SHAP has been applied widely to liver disease classification. Almusallam and Khan (2025) [34] applied SHAP for feature selection in a DNN liver disease classifier using the Indian Liver Patient Dataset. Fan et al. (2023) [35] developed the IHCP system combining SHAP global explanations with a stability-enhanced LIME variant (LIME_stability) for HCV prediction, published in *BMC Bioinformatics*; this work informed the design of our inter-method overlap analysis. Ali et al. (2023) [36] applied SHAP to five classifiers for HCV diagnosis on a Jordanian hospital dataset of 1801 patients; enzyme markers ranked consistently across all five. Khan et al. (2025) [37] integrated SHAP and LIME into a multi-model liver disease prediction framework and demonstrated that a high-performing ensemble boosting classifier and XAI interpretability can be achieved together. Despite this body of work, no prior HCV study has applied a formal rank correlation measure to quantify cross-model SHAP agreement—the principal methodological contribution of the present work.

### 1.4. Explainable AI: LIME

LIME, proposed by Ribeiro et al. (2016) [15] at KDD, learns an interpretable local linear surrogate around individual predictions by generating a perturbed neighbourhood of each instance, obtaining black-box predictions for all perturbed samples, and fitting a weighted sparse linear model. Being entirely model-agnostic, LIME produces directional feature weights that clinicians can read as factors pushing a prediction toward or away from a particular class. Hassan et al. (2024) [38] reviewed 52 articles on LIME in medical imaging and concluded that LIME explanations helped clinicians interpret model decisions and raised confidence in those decisions across the studies surveyed. Sathyan et al. (2022) [39] showed experimentally that LIME and SHAP capture different things—SHAP reflects overall feature importance, LIME the local decision behaviour—and when the two agree, the biomarker signal is on firmer ground than it would be from either method alone. LIME does have a weakness. Because it draws a fresh random perturbation sample on each call, repeated runs can return different rankings for the same instance [16], and this gets worse when class imbalance distorts the local neighbourhood. To limit that sensitivity, we do not rely on LIME alone but compare its top-5 local features with the corresponding SHAP top-5 across all model–class combinations.

### 1.5. Counterfactual Explanations and DiCE

Counterfactual explanations answer the clinical question: what minimal changes to biochemical markers would shift a predicted stage to a more favourable outcome? Mothilal et al. (2020) [31] introduced DiCE (Diverse Counterfactual Explanations) at ACM FAT*, addressing two prior limitations: lack of diversity in generated counterfactuals and the absence of feasibility constraints. DiCE adds a diversity penalty to the optimisation objective so that the solutions it returns are meaningfully different from each other, and allows the user to set bounds on how far each feature can move. In the HCV context, this enables generating potentially informative what-if scenarios—for example, what changes in the biochemical markers would be sufficient to shift a Cirrhosis prediction toward Donor/Control, and whether these changes are consistent with biochemical responses observed during successful DAA therapy [3]. A 2025 systematic review [40] identified counterfactual explanations as particularly useful for treatment planning and personalised medicine, given their capacity to frame predictions as answerable what-if questions. However, the review identifies clinical validation with predefined feature-range constraints as an open problem, which informed our decision to apply permitted_range bounds throughout the DiCE analysis.

### 1.6. Cross-Model XAI Agreement

An explanation is only clinically trustworthy if it survives a change of model, and few studies test whether it does. Consider two models with matching accuracy but conflicting importance rankings: which one tells the clinician about the biology? There is no way to know. Rai et al. (2025) [16] made this concrete, testing SHAP and LIME across levels of class imbalance on UK CPRD lung-cancer data; both grew significantly less consistent as the imbalance deepened (*p* < 0.01, Wilcoxon signed-rank test). Given that Donor/Control instances outnumber Fibrosis instances by roughly 26:1 in the UCI HCV dataset [23], this degradation pattern is a concrete concern for the present analysis. Hermosilla et al. (2025) [17] found stability scores of 0.89 for XGBoost versus 0.72 for TabNet—a difference they attribute to architecture rather than data. On general tabular data, XGBoost outperforms deep neural network architectures [41]; on tabular medical datasets specifically, both XGBoost and LightGBM achieve the highest average ranks among compared methods [42], suggesting their classification advantage may extend to explanation stability as well. To our knowledge, no prior study has applied Spearman rank correlation to formally quantify pairwise cross-model SHAP agreement for HCV staging, nor has any study reported SHAP-LIME overlap heatmaps across all model–class combinations for this domain.

We address these gaps through five contributions. First, we build a multi-model XAI pipeline spanning five ML architectures (LR, RF, XGBoost, LightGBM, SVM) under a preprocessing protocol designed to minimise data leakage. Second, we introduce a cross-model SHAP agreement analysis using Spearman rank correlation that formally quantifies how consistently features are ranked across architectures. Third, we compute stage-specific SHAP profiles for each disease class, tracing the biochemical gradient from healthy controls to end-stage Cirrhosis. Fourth, we conduct a systematic SHAP-LIME overlap analysis across all 20 model–class combinations. Fifth, we generate DiCE counterfactual explanations within predefined feature-range bounds for representative disease-stage patients. Predictive performance was evaluated using both held-out testing and 5-fold stratified cross-validation with preprocessing applied independently within each fold.

## 2. Materials and Methods

### 2.1. Dataset

**Figure 1:**
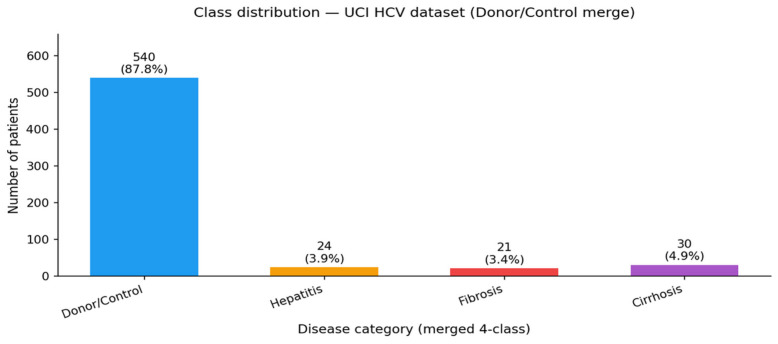
UCI HCV dataset class distribution after merging the suspect blood-donor records into Donor/Control (four classes, *n* = 615). Donor/Control records constitute 87.8% of the cohort, motivating SMOTE and macro-averaged evaluation metrics.

**Figure 2:**
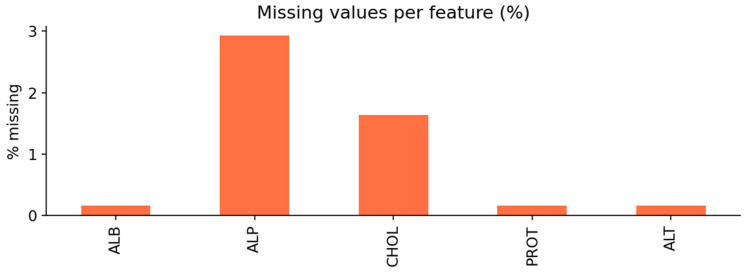
Missing value rates per feature in the UCI HCV Dataset. ALP contains the highest proportion of missing values (2.9%), followed by CHOL (1.6%), while ALB, PROT, and ALT each contain approximately 0.2% missing data.

The UCI HCV dataset (id = 571) [23] comprises 615 patient records with 12 features: Age, Sex, and 10 biochemical laboratory markers (ALB, ALP, ALT, AST, BIL, CHE, CHOL, CREA, GGT, PROT). The dataset originates from a machine learning study on HCV-related liver Fibrosis conducted at the Medical University Hannover, Germany [43], before being expanded and deposited in the UCI repository [23]. The 7 suspect blood-donor records (originally 1.1% of the cohort) were merged with the 533 blood-donor records into a single Donor/Control class, since a 7-instance class averages roughly one instance per validation fold and yields statistically unreliable per-class estimates. The resulting four target classes are Donor/Control (*n* = 540, 87.8%), Hepatitis (*n* = 24, 3.9%), Fibrosis (*n* = 21, 3.4%), and Cirrhosis (*n* = 30, 4.9%). Missing values are present in five features: ALP (2.9%), CHOL (1.6%), ALB (0.2%), PROT (0.2%), and ALT (0.2%). The class distribution appears in Figure 1; missing value rates per feature are summarised in Figure 2.

### 2.2. Leakage-Controlled Preprocessing Pipeline

Preventing data leakage requires applying preprocessing in a strict split-first order. Skipping this step is a well-documented source of optimistic bias in published medical ML studies, with data leakage affecting at least 294 studies across 17 scientific fields and, in some cases, leading to performance claims that disappear entirely when errors are corrected [44]. The four-step sequence is (1) stratified 80/20 train/test split (492 training, 123 test instances), preserving class proportions; (2) median imputation fitted exclusively on the training set and applied to both sets, preventing test-set distribution information from influencing imputed values. Median imputation was chosen over mean imputation for its robustness to the skewed biochemical distributions, and over model-based schemes such as KNN imputation to keep the pipeline transparent and avoid inserting a second learned model into each fold. The imputer was refitted on each training fold, so no validation-fold statistics reached the imputed values. (3) StandardScaler was fitted on the training set alone, and the test data were standardised with training statistics only [45]. (4) SMOTE ran on the scaled training set. Merging the suspect-donor records into Donor/Control raised the smallest minority class (Fibrosis) to 21 records, enough for the default k_neighbors = 5; the original 7-instance class had forced k = 1, so this setting could now be used. Under 5-fold stratified cross-validation, each of the four steps ran independently inside every fold. SMOTE is widely used to address class imbalance in medical datasets [46], though how well it works depends on the k_neighbors setting and how severe the imbalance is [46]. After SMOTE, the training set contains equal numbers of instances per class.

### 2.3. Model Training

Five classifiers with fixed hyperparameters were selected to cover a range of architectural inductive biases. Logistic Regression (L2 regularisation, max_iter = 2000) served as a linear baseline whose additive structure produces a distinct feature importance profile. Random Forest (n_estimators = 100, Gini impurity) [47] aggregates predictions from an ensemble of decision trees through majority voting, capturing non-linear feature interactions. XGBoost (n_estimators = 100, mlogloss objective) [48] builds depth-wise gradient-boosted trees with L1/L2 regularisation and is well established on tabular data [41]. LightGBM (n_estimators = 100, leaf-wise growth) uses a histogram-based boosting strategy with leaf-wise tree growth to capture high-order feature interactions efficiently [42]. The SVM (RBF kernel, C = 1.0) [49] draws its decision boundary at the widest margin in the kernel-projected space. We standardised the inputs first, as SVMs are sensitive to feature scale [45]. Hyperparameters were held fixed by design: with tuning removed, any divergence in the feature-importance rankings has to come from the architecture itself. The complete set of final hyperparameter values for every model, together with the tuning strategy, is reported in Table 1 to support reproducibility. On tabular datasets, XGBoost outperforms deep neural network approaches [41], and both XGBoost and LightGBM rank highest across tabular medical diagnosis benchmarks [42]. Evaluation uses accuracy, macro-averaged F1, and macro-averaged one-vs-rest AUC-ROC on both the held-out test set and 5-fold CV.

### 2.4. SHAP Explainability

SHAP analysis is applied at three levels. For RF, XGBoost, and LightGBM, TreeExplainer exploits the model’s internal tree structure to compute Shapley values exactly [14], without the sampling approximations that kernel-based methods require. For LR and SVM, KernelExplainer was used with 30 k-means background samples drawn from the training distribution (the standard SHAP library default) [14]. To place all models on an equal footing, all five models, tree-based ones included (via TreeExplainer), were explained on one fixed 50-instance subset of the scaled test set. Every cross-model Spearman comparison then used the same instances rather than different sample sizes. Global feature importance is the mean absolute SHAP value across samples and classes, computed as np.abs(sv).mean(axis = (0, 2)); here sv is the 3D array of shape (n_samples, n_features, n_classes) returned by current SHAP versions. For per-class importance we slice sv[:,:,class_idx], which lets us track, stage by stage, the features each model leaned on. Beeswarm plots for the Hepatitis class show whether high or low feature values push predictions toward that class, giving a directional picture of each feature’s role.

### 2.5. LIME Explainability

LimeTabularExplainer [15] was used with the following configuration: the SMOTE-augmented training data served as the background dataset; 5000 perturbation samples were generated per explanation; and a representative test instance (the sample closest to each class centroid in standardised feature space) was selected per disease class. LIME explanations were generated for the target disease-stage label of each representative instance (labels = [cls_idx]) rather than the model’s top predicted class, ensuring that each explanation corresponds to that labelled disease stage regardless of model prediction. Top-5 LIME feature weights (by absolute magnitude) were compared against top-5 SHAP features per model–class pair, with overlap defined as the count of features appearing in both lists (0–5 scale). Across five models and four disease stages, this yields 20 model–class combinations in total. This overlap analysis measures consistency between global SHAP rankings and representative local LIME explanations—it is a practical heuristic, not a formal equivalence test between the two methods.

### 2.6. DiCE Counterfactuals

DiCE [31] was applied with permitted_range constraints (±3.5 standardised units) to reduce extreme counterfactual values. Age and Sex were declared immutable through the features_to_vary argument, so DiCE was permitted to alter only the ten modifiable biochemical markers. Demographic attributes are not clinically modifiable, so constraining the search this way makes the counterfactuals more clinically interpretable. The permitted_range setting constrains the final counterfactual feature values, not the size of the change from the patient’s original values. When a patient’s original value lies far outside the interval, the resulting change can be large even though the final value is bounded. LightGBM was chosen as the DiCE base model because it achieved the highest macro-F1 in the main 5-fold cross-validation run and stayed closely matched with XGBoost under repeated-seed cross-validation. Four diverse counterfactuals were generated per query instance using the random method. Representative query patients were drawn from held-out test instances whose standardised feature values lay within the same ±3.5 SD range used for counterfactual generation, so that the illustrations reflect patients inside the constrained search space rather than extreme outlier profiles. In practice, this constraint acts as a soft target rather than a hard clamp—most final feature values fall within the stated bounds, but the random method can occasionally produce marginal exceptions, as noted in Section 4.5. Representative Cirrhosis and Fibrosis patients were queried with Donor/Control or Hepatitis as target classes.

### 2.7. Cross-Model SHAP Agreement Analysis

For all 10 model pairs formed from the five classifiers, Spearman rank correlation (rho) is computed between the global SHAP importance vectors—the mean absolute SHAP values per feature averaged over samples and classes. Spearman correlation suits this better than Pearson. Pearson would assume the importance scores rise together linearly across architectures, which is not the question here; the question is whether the feature ordering is preserved, regardless of how the magnitudes scale. At rho close to 1.0, two models place all 12 features in nearly the same order; near 0, their orderings barely relate. Run over every pair of models, this tells us whether the explanations group by architecture and exposes any model that ranks features unlike the rest.

### 2.8. Reproducibility

All experiments were run in Python 3.12.13 with scikit-learn 1.6.1, XGBoost 3.3.0, LightGBM 4.6.0, SHAP 0.52.0, LIME 0.2.0.1, and DiCE (dice-ml 0.12), and the full pipeline is available as a Google Colab notebook. We fixed the random seed (RANDOM_STATE = 42) everywhere except the DiCE step, where the installed version did not support a fixed random seed. We also repeated the 5-fold evaluation over five random seeds to check how stable the rankings were; those results sit in Section 3.1 and are taken up again under external validation in Section 4.5.

## 3. Results

### 3.1. Model Performance

**Figure 3:**
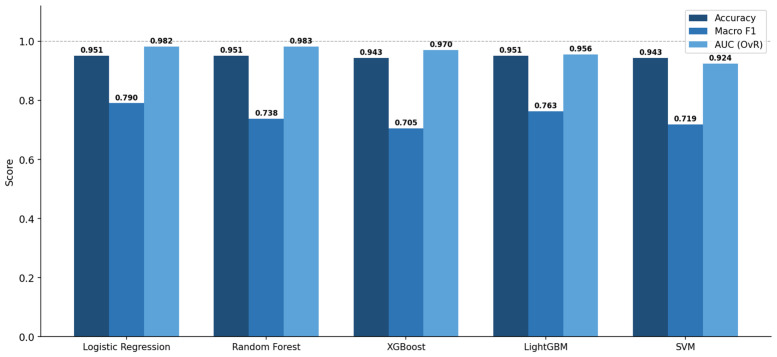
Model performance comparison—held-out test set. Note: CV results (Table 3) show a different minority-class ranking. LightGBM achieves the best CV macro-F1 (0.684), while Logistic Regression, the strongest model on the single test split, drops to 0.598 under CV.

**Figure 4:**
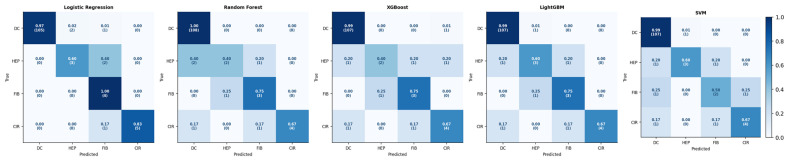
Normalised confusion matrices—all five models (test set). DC = Donor/Control, HEP = Hepatitis, FIB = Fibrosis, CIR = Cirrhosis.

**Figure 5:**
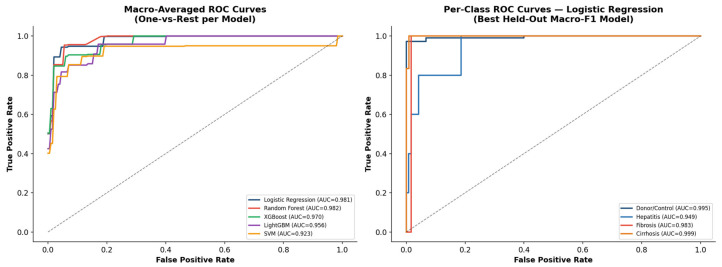
ROC curves. (**Left**): Macro-averaged one-vs-rest. (**Right**): Per-class for Logistic Regression, the best held-out macro-F1 model. All models achieve macro-AUC above 0.92, with Logistic Regression and Random Forest highest (0.9815 and 0.9825, shown as 0.981 and 0.982 in the figure legend). The right panel shows Logistic Regression per-class AUC values of 0.995 (Donor/Control), 0.949 (Hepatitis), 0.983 (Fibrosis), and 0.999 (Cirrhosis). The dashed diagonal marks the no-discrimination reference (chance level, AUC = 0.5).

Table 2 reports held-out test performance, Table 3 the 5-fold cross-validation results (mean ± std), and Figure 3 the comparison. On the held-out set, every model cleared 94% accuracy. Three of them tied for the top accuracy of 0.9512 (Logistic Regression, Random Forest, LightGBM). On macro-F1, Logistic Regression led at 0.7904, then LightGBM at 0.7632 and Random Forest at 0.7382; SVM and XGBoost followed at 0.7189 and 0.7049, respectively. The confusion matrices in Figure 4 show that these headline numbers hide a lot of per-class variation, and macro-AUC-ROC spanned 0.9239 for SVM to 0.9825 for Random Forest, with Logistic Regression a fraction behind at 0.9815 (Figure 5 reports these to three decimals). The merged Donor/Control class was classified near-perfectly by all five models (recall 0.97–1.00). Hepatitis remained difficult: Logistic Regression, LightGBM and SVM reached 0.60 recall (three of five instances), while Random Forest and XGBoost recovered 0.40 (two of five), with the misclassifications scattered toward Donor/Control and Fibrosis. Fibrosis was recovered best by Logistic Regression, which classified all four test instances correctly; Random Forest, XGBoost and LightGBM each recovered three of four (0.75 recall), while SVM recovered two. For Cirrhosis, Logistic Regression reached 0.83 recall (five of six correct) while the remaining four models recovered four of six (0.67); the misclassified instances fell into Fibrosis or the neighbouring Donor/Control class. The per-class ROC curves for Logistic Regression (Figure 5, right panel), the best test-set model, show AUC values of 0.995 for Donor/Control, 0.949 for Hepatitis, 0.983 for Fibrosis, and 0.999 for Cirrhosis. Under 5-fold stratified cross-validation (Table 3), macro-F1 scores fell to 0.598–0.684, below the single-split figures, and standard deviations of 0.031–0.086 across folds point to real instability in minority class results. LightGBM achieved the best CV macro-F1 (0.6840 ± 0.0311), while Logistic Regression dropped from 0.790 on the test split to 0.598 ± 0.063 under CV. LightGBM achieved the highest mean CV accuracy (0.9463), while Random Forest led on CV AUC-ROC (0.9652 ± 0.0118); Random Forest and LightGBM were the most stable in terms of CV accuracy variance (±0.0083 for LightGBM and ±0.0065 for Random Forest). To check that these rankings are not an artefact of a single fold assignment, the 5-fold evaluation was repeated across five random seeds. Re-seeding rearranged little at the top. XGBoost and LightGBM again led and again sat close, at 0.648 ± 0.023 and 0.645 ± 0.026, followed by Random Forest (0.631 ± 0.030), SVM (0.604 ± 0.024), and Logistic Regression (0.591 ± 0.019). So neither the Logistic Regression result on the single split nor the LightGBM result in the one 5-fold run deserves much weight; repeat the resampling and the strongest tree models converge.

### 3.2. Global SHAP Feature Importance

**Figure 6:**
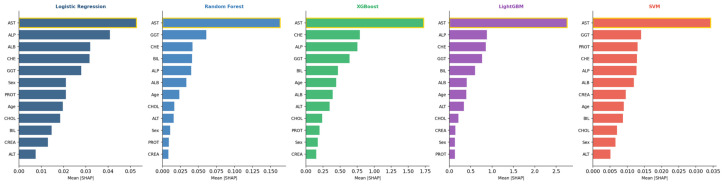
Global SHAP feature importance—all five models. Gold border marks top-ranked feature per model. AST ranks first across all five models. In LR, ALP, ALB, CHE and GGT follow AST, sharing four of the top five features with the tree-based consensus.

Figure 6 presents global SHAP feature importance for all five models. AST ranks first across all five models. Among the tree-based models, the next most important features are GGT and CHE for Random Forest, CHE and ALP for XGBoost, and ALP and CHE for LightGBM, so AST, CHE, GGT and ALP recur throughout the tree-based top-five lists. SVM shows a partially similar profile, with AST, GGT, and ALP among its top-ranked features, although PROT and CHE also appear prominently. Logistic Regression aligns on the leading markers as well: AST ranks first, followed by ALP, ALB, CHE, and GGT, sharing four features (AST, ALP, CHE, GGT) with the tree-based consensus. The correlation values between LR and the tree-based models (rho = 0.622–0.643) confirm this partial but real alignment. 

### 3.3. SHAP Beeswarm Plots (Hepatitis Class)

**Figure 7:**
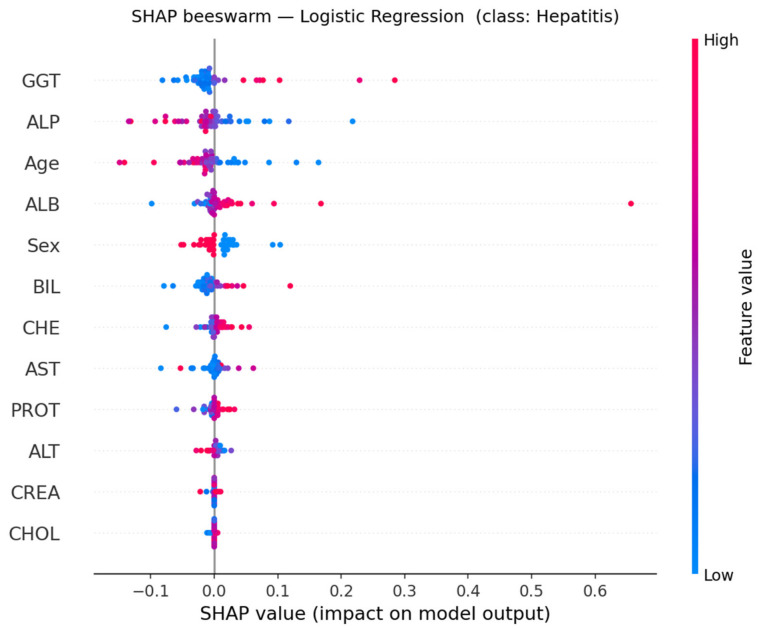
SHAP beeswarm—Logistic Regression (Hepatitis class). GGT, ALP and Age show a wide spread; most other features cluster near zero.

**Figure 8:**
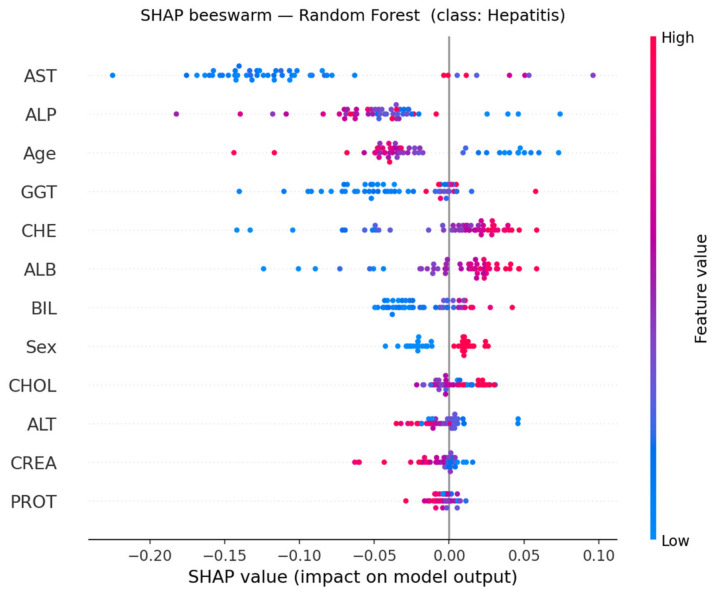
SHAP beeswarm—Random Forest (Hepatitis class). AST shows strong bidirectional effects.

**Figure 9:**
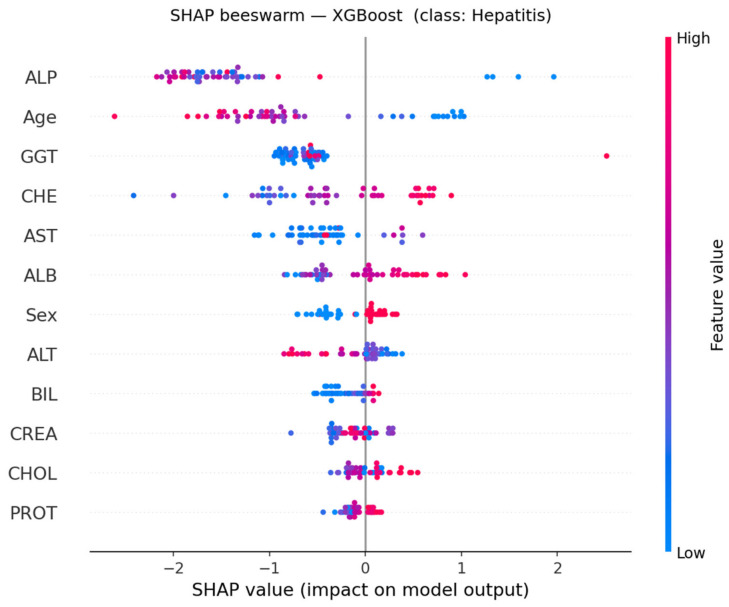
SHAP beeswarm—XGBoost (Hepatitis class). ALP ranks first, with Age and GGT also showing wide spread.

**Figure 10:**
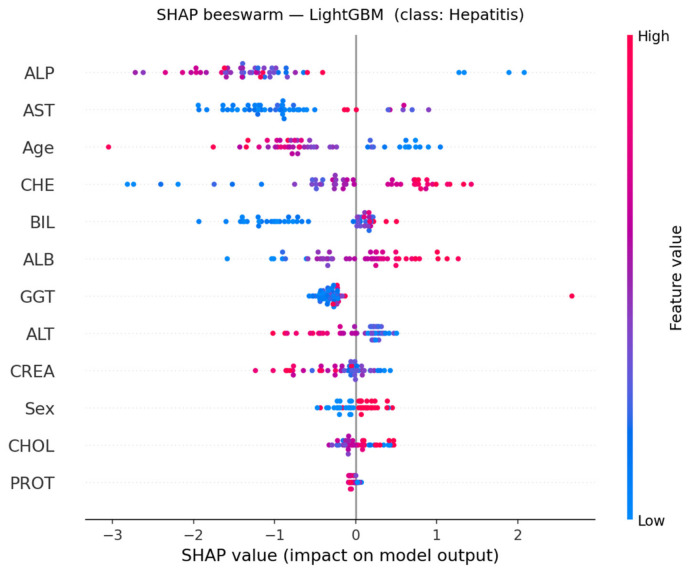
SHAP beeswarm—LightGBM (Hepatitis class). ALP ranks first, followed by AST and Age.

**Figure 11:**
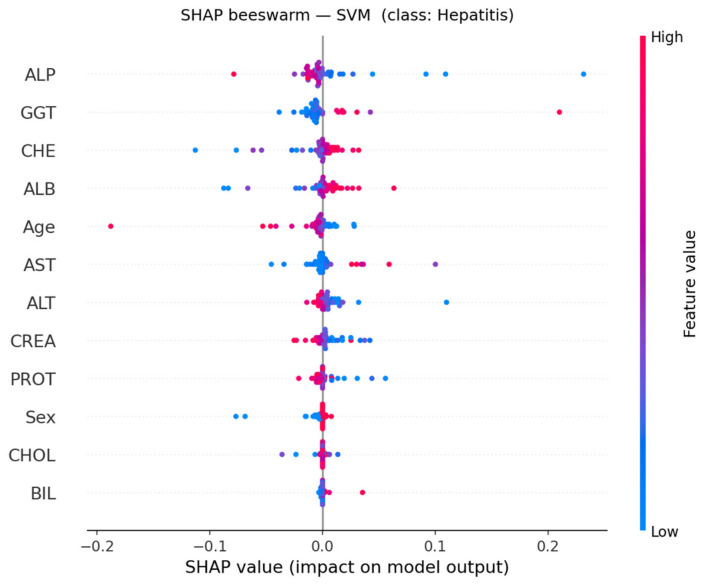
SHAP beeswarm—SVM (Hepatitis class). ALP, GGT and CHE dominate.

Figure 7, Figure 8, Figure 9, Figure 10 and Figure 11 show SHAP beeswarm plots for the Hepatitis class. Across all models, low AST (blue) produces negative SHAP contributions while high AST pushes toward Hepatitis. Higher ALB values push predictions toward Hepatitis in all models, reflecting the role of albumin as a marker of intact hepatic synthetic function [5]. Model-specific patterns differ in both feature ranking and SHAP magnitude. For Logistic Regression (Figure 7), GGT, ALP and Age show the widest spread, with higher Age associated with negative SHAP contributions toward Hepatitis; most other features cluster tightly near zero, reflecting the model’s linear structure (SHAP range approximately −0.15 to +0.65). This feature ordering reflects SHAP values computed for the Hepatitis class specifically, so it differs from the global importance ranking in Figure 6, where AST ranks first for LR overall. For Random Forest (Figure 8), AST is the dominant feature with strong bidirectional effects: low AST values (blue) produce strongly negative contributions reaching approximately −0.20, while high AST (red) pushes positively; ALP and Age also show a wide spread. For XGBoost (Figure 9), ALP ranks first with the widest spread, and high ALP values (red) generate negative SHAP contributions, pushing away from the Hepatitis class; Age ranks second with a broad spread reaching roughly −2 SHAP units, and GGT completes the leading three features. For LightGBM (Figure 10), ALP ranks first, followed by AST and Age, and the overall SHAP range (approximately −3 to +3) is wider than the linear models, consistent with the larger SHAP magnitudes often observed in boosted tree ensembles. For SVM (Figure 11), ALP, GGT and CHE are the dominant features but overall SHAP values remain tightly distributed (range approximately −0.2 to +0.25), expected behaviour for an RBF-kernel SVM operating in standardised feature space.

### 3.4. Stage-Specific SHAP Analysis

**Figure 12:**
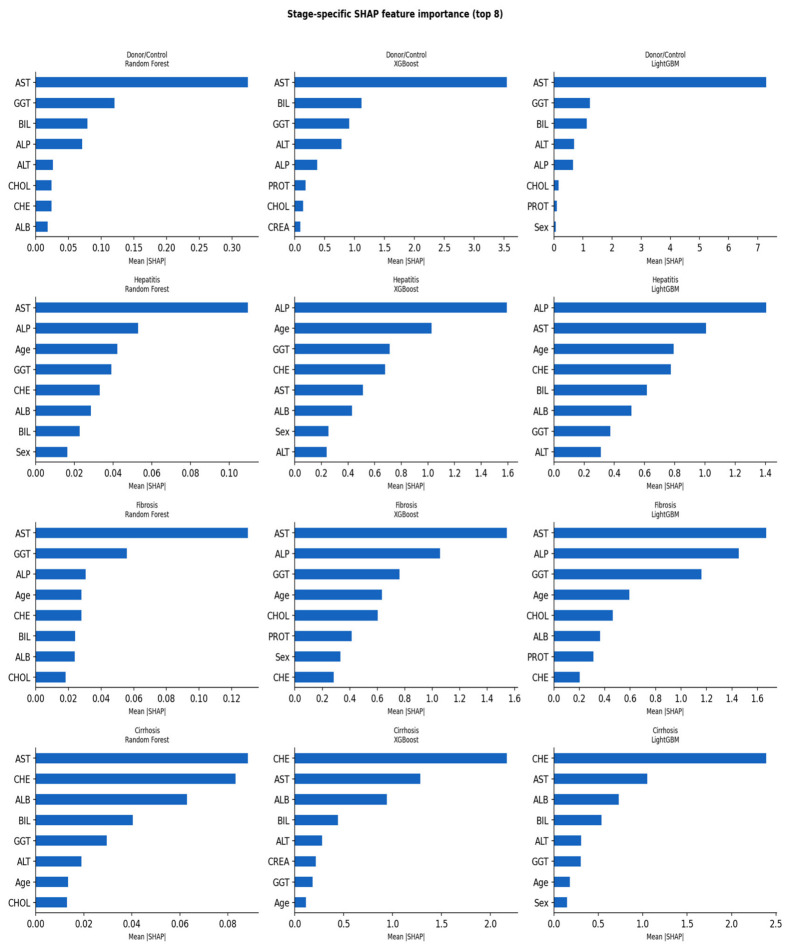
Stage-specific SHAP feature importance (top 8 per stage, tree-based models). CHE leads Cirrhosis-stage importance in XGBoost and LightGBM and ranks second in Random Forest, in line with established biochemical patterns of end-stage liver disease in HCV.

Figure 12 presents stage-specific SHAP importance (top-8 per stage, tree-based models). The feature rankings shift in a way that tracks disease severity: AST and GGT dominate at Donor/Control; ALP and Age become prominent at Hepatitis; AST, ALP, and GGT co-dominate at Fibrosis; and CHE rises to the top at Cirrhosis. Most striking of all, CHE ranks first for Cirrhosis in XGBoost and LightGBM and second, behind AST, in Random Forest, reflecting end-stage hepatic synthetic failure [6,7,8]. At the Donor/Control stage, AST ranks first in all three tree-based models, with GGT and BIL taking the next positions (GGT second for Random Forest and LightGBM, BIL second for XGBoost); cross-model agreement at this stage is high. For Hepatitis, ALP and AST occupy the leading positions: XGBoost and LightGBM rank ALP first while Random Forest ranks AST first, and Age appears in the top four for all three models. For Fibrosis, AST, ALP, and GGT all appear in the top four across the three models, with Age and CHOL also present in multiple rankings. For Cirrhosis, CHE ranks first in XGBoost and LightGBM and second in Random Forest, where AST ranks first; ALB ranks among the top three in all three models, and BIL, AST, and ALT are consistently present in the top eight. Overall, the models agree most strongly at the two ends of the spectrum, Donor/Control (where AST and GGT dominate) and Cirrhosis (where CHE and AST dominate), with more variability at the intermediate stages.

### 3.5. Cross-Model SHAP Agreement

**Figure 13:**
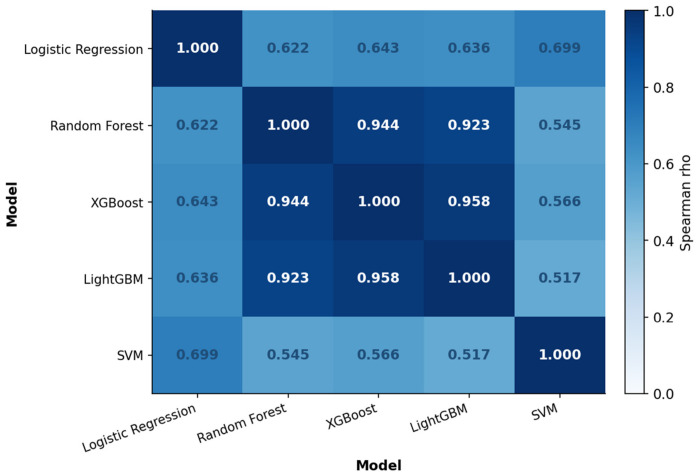
Cross-model SHAP agreement—Spearman rank correlation. Tree-based models: rho = 0.923–0.958. LR vs. tree-based models: rho = 0.622–0.643. LR–SVM: rho = 0.699.

Figure 13 is the Spearman rank-correlation heatmap. Three of the models, Random Forest, XGBoost, and LightGBM, form a dark cluster, correlating at 0.944, 0.923, and 0.958 across the three tree–tree pairs. The other two stand outside it. Logistic Regression and SVM agree with each other at 0.699 and with the tree models by less: SVM lands between 0.517 and 0.566, Logistic Regression between 0.622 and 0.643. Taking the cross-block pairs from weakest up: LightGBM–SVM 0.517, RF–SVM 0.545, XGBoost–SVM 0.566, LR–RF 0.622, LR–LightGBM 0.636, LR–XGBoost 0.643, LR–SVM 0.699. Logistic Regression, then, resembles XGBoost most and Random Forest least among the tree models. 

### 3.6. LIME Local Explanations

**Figure 14:**
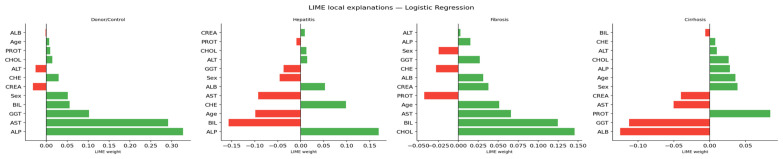
LIME—Logistic Regression (all 4 classes). Green bars indicate features contributing toward the predicted class (positive LIME weight) and red bars indicate features contributing against it (negative weight).

**Figure 15:**
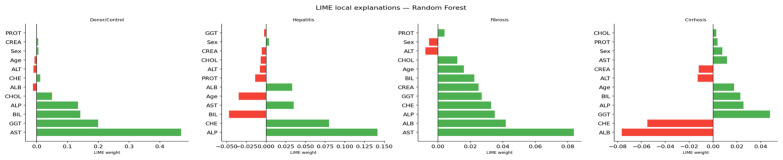
LIME—Random Forest (all 4 classes). Green bars indicate features contributing toward the predicted class (positive LIME weight) and red bars indicate features contributing against it (negative weight).

**Figure 16:**
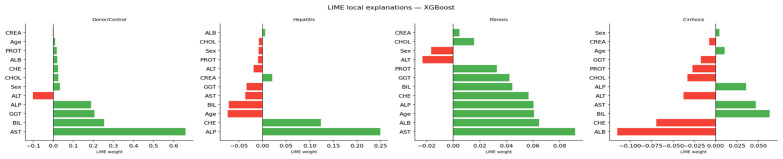
LIME—XGBoost (all 4 classes). Green bars indicate features contributing toward the predicted class (positive LIME weight) and red bars indicate features contributing against it (negative weight).

**Figure 17:**
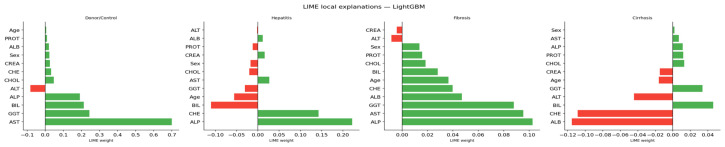
LIME—LightGBM (all 4 classes). GGT and BIL are the only two features with positive LIME weight among LightGBM’s Cirrhosis top-5; ALB and CHE, both negative, are larger in magnitude. Green bars indicate features contributing toward the predicted class (positive LIME weight) and red bars indicate features contributing against it (negative weight).

**Figure 18:**
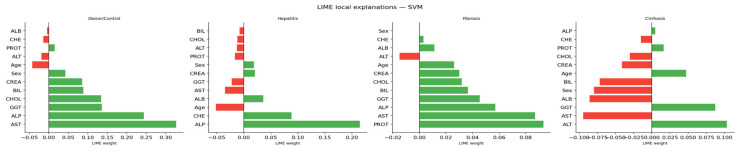
LIME—SVM (all 4 classes). Green bars indicate features contributing toward the predicted class (positive LIME weight) and red bars indicate features contributing against it (negative weight).

Figure 14, Figure 15, Figure 16, Figure 17 and Figure 18 present LIME bar charts for all five models across the four disease classes. CHE appears among the top LIME features for Cirrhosis in the three tree-based models and carries a strong weight in each of them. This agreement carries weight because SHAP and LIME derive explanations through different computational mechanisms. For the Donor/Control representative instance, AST carries the largest positive LIME weight in four of the five models, with ALP marginally ahead of AST in Logistic Regression; the second-largest positive weight is taken by GGT, ALP, or BIL depending on the architecture; ALP and BIL also contribute positively in several models. For the Hepatitis representative instance, ALP carries the strongest positive weight across all five models, with CHE ranking second in the tree-based models and SVM, while Age contributes negatively across all five models. For the Fibrosis representative instance, AST, ALP, and ALB show prominent positive weights in most models, and the tree-based models place GGT among the leading features. For the Cirrhosis representative instance, GGT carries a positive LIME weight in Random Forest, LightGBM, and SVM but a negative one in Logistic Regression and XGBoost, and BIL a positive weight in Random Forest, XGBoost, and LightGBM but a negative one in SVM, so neither marker is uniformly positive across models. ALB appears as a negative weight in all five models, and CHE as a negative weight in Random Forest, XGBoost, and LightGBM, indicating that in these representative instances the local surrogate places them against the Cirrhosis label. The consistent negative ALB weight across every model is the clearest single cross-model signal at this stage. Several models additionally display negative LIME weights (red bars), representing features that act against the predicted class for that representative instance; examples include CREA for Logistic Regression and AST for SVM at Cirrhosis.

### 3.7. SHAP-LIME Feature Overlap

**Figure 19:**
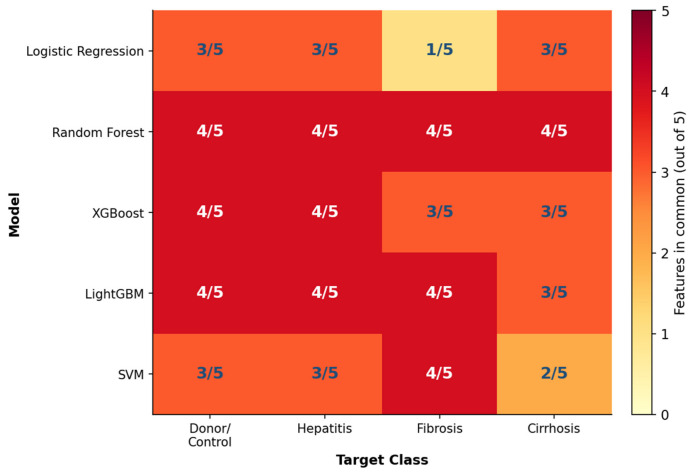
SHAP-LIME top-5 feature overlap (per model × class). Ranges from 1/5 (LR, Fibrosis) to 4/5 (Random Forest on all four classes; LightGBM on Donor/Control, Hepatitis, and Fibrosis; XGBoost on Donor/Control and Hepatitis).

The SHAP-LIME top-5 overlap from the run is shown in Figure 19, and it spans 1/5 to 4/5. The floor, 1/5, belongs to LR on Fibrosis: here the global SHAP ranking and the local LIME explanation scarcely coincide. At the other end, Random Forest reaches 4/5 on every class, XGBoost on Donor/Control and Hepatitis, LightGBM on Donor/Control, Hepatitis, and Fibrosis; Table 4 lays out all 20 cells. The per-model averages are 2.50/5 (Logistic Regression), 3.00/5 (SVM), 3.50/5 (XGBoost), 3.75/5 (LightGBM), and 4.00/5 (Random Forest), with the lowest agreement with LIME for Logistic Regression and highest for Random Forest. By class, Cirrhosis averages 3.0/5, Fibrosis 3.2/5, and Donor/Control and Hepatitis 3.6/5 apiece. Donor/Control and Hepatitis show the highest average SHAP-LIME agreement across models, while Cirrhosis shows the lowest, driven mainly by the SVM 2/5 result. The clearest cross-method finding concerns CHE at the Cirrhosis stage: CHE appears in the LIME top-5 for Cirrhosis in three of the five models, all of them tree-based, and in the global SHAP top-5 for all five models. Because the SHAP ranking is global while the LIME ranking is class-specific, this is best read as partial cross-method convergence on CHE as a Cirrhosis marker rather than exact feature-class equivalence. In this analysis, CHE for Cirrhosis was the clearest cross-method finding, particularly among the tree-based models. 

### 3.8. DiCE Counterfactual Explanations

**Figure 20:**
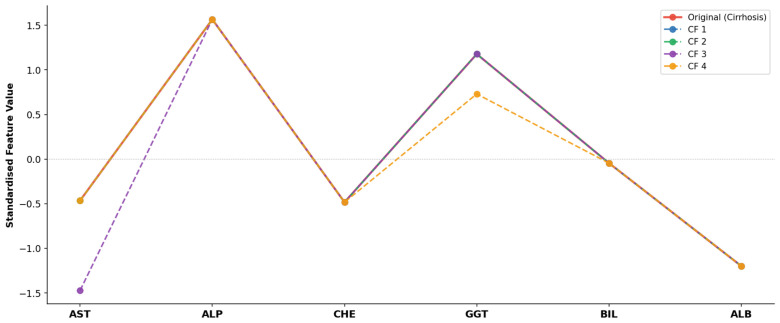
DiCE counterfactual explanations—cirrhosis patient (4 counterfactuals targeting Donor/Control; Age and Sex fixed). Solid red: original Cirrhosis patient. Dashed: the four counterfactuals, with Age and Sex held immutable. Among the six plotted markers, CF3 reaches the Donor/Control class mainly through a large AST reduction and CF4 mainly through a GGT reduction, while CF1 and CF2 coincide with the original profile here and move only the four features not shown (ALT, CHOL, CREA, PROT). This figure visualises the four individual DiCE solutions for this single Cirrhosis-to-Donor/Control query, whereas Table 5 summarises only the most-changed features for this and the additional representative queries (Patients B and C). Values in standardised units.

With Age and Sex held immutable, the DiCE analysis revealed distinct counterfactual profiles for each patient built entirely from modifiable biochemical markers: Patient A’s Cirrhosis-to-Donor/Control shift was driven by ALB and BIL elevation; Patient B’s Cirrhosis-to-Hepatitis shift required ALB, AST, and GGT increases; and Patient C’s Fibrosis-to-Donor/Control shift was characterised by an ALP rise and a modest BIL reduction. As illustrated in Figure 20 and Table 5, the LightGBM-based counterfactual explanations indicated that changes in ALB, BIL, AST, GGT, and ALP values were associated with transitions from Cirrhosis or Fibrosis toward the Donor/Control or Hepatitis classes. Figure 20 displays the four diverse counterfactuals generated for the Cirrhosis patient targeting the Donor/Control class, plotted in standardised feature space across six features (AST, ALP, CHE, GGT, BIL, ALB), with Age and Sex fixed and therefore omitted from the plot. All four counterfactuals (CF1–CF4, dashed lines) achieve the Donor/Control prediction through different routes. Across the six plotted markers, CF1 and CF2 coincide with the original profile, reaching the target class by altering only the four mutable features not shown in the plot (ALT, CHOL, CREA, and PROT). CF3 differs from the original in a single displayed feature, reducing AST to roughly −1.5 SD, while CF4 lowers GGT most strongly; ALP, CHE, BIL, and ALB remain essentially unchanged across all four solutions. The spread of counterfactual solutions indicates that the LightGBM model does not enforce a single fixed biochemical route to the Donor/Control class; the supporting feature changes vary considerably across the four solutions. Because DiCE was run without a fixed seed and Figure 20 and Table 5 come from separate calls (Section 2.8 and Section 4.5), the specific features moved in this figure differ from the summary in Table 5, which is drawn from an independent counterfactual set for the same query. Table 5 reports the most-changed modifiable features per patient query; the complete four-counterfactual profiles for the Cirrhosis-to-Donor/Control query are shown in Figure 20. For example, Patient A was associated with a model prediction shift requiring an ALB increase of +2.461 SD (from −1.198 SD to +1.263 SD) together with a BIL increase of +2.176 SD to achieve a Donor/Control prediction. Patient B’s prediction shift was associated with substantial ALB elevation (+3.616 SD), AST elevation (+2.706 SD), and GGT elevation (+2.240 SD) to move from Cirrhosis toward Hepatitis; Patients A and B share the same original ALB value (−1.198 SD), suggesting they represent the same Cirrhosis patient queried with two different target classes. Patient C’s prediction shift involved an ALP increase (+2.960 SD) and a modest BIL reduction (−0.699 SD), with Age and Sex held fixed throughout. Several of the dominant counterfactual changes, particularly the recovery of ALB and the changes in AST and GGT, are directionally consistent with known biochemical patterns in HCV progression. 

### 3.9. Computational Cost of the XAI Methods

We timed each XAI computation on the shared 50-instance subset to gauge deployability (Table 6). The spread is wide, and it tracks how the explainers work. TreeExplainer cleared the tree-based models in 0.10 to 0.37 s. KernelExplainer, which handles the model-agnostic explanations for Logistic Regression and SVM, was slow by comparison: 5.96 s for Logistic Regression, and 102.49 for SVM. That SVM figure was the heaviest computation in the whole pipeline and would dominate any real-time budget. LIME took roughly 0.07 s for one instance at 5000 perturbation samples, and DiCE about 0.22 s for four counterfactuals on a single query. The upshot: tree-based SHAP, LIME, and DiCE are all fast enough for interactive use, and only kernel-based SHAP on the non-tree models would need a faster approximation to scale.

## 4. Discussion

### 4.1. Single-Split vs. Cross-Validation: A Critical Methodological Finding

The gap between single-split and cross-validation performance is one of the clearer results here. Logistic Regression tops the held-out split at macro-F1 0.790 but falls to 0.598 ± 0.063 under 5-fold CV, about 19 percentage points, and the tree-based models swap ranks in much the same way between the two settings. LightGBM has the best CV macro-F1 (0.6840 ± 0.0311), even though its test-set accuracy (95.1%) only ties several other models. Its leaf-wise boosting and built-in regularisation (min_child_samples, lambda) [42] may explain the robustness when minority classes are re-sampled repeatedly, but we did not test that directly. The gap traces back to the extreme minority class imbalance. With only 21 Fibrosis, 24 Hepatitis, and 30 Cirrhosis instances across the whole dataset, any single stratified 80/20 split can produce minority class compositions in the test set that happen to favour one architecture over another. Averaging over five stratified validation folds, the CV results give a more dependable picture of how well each model will generalise, and the repeated-seed analysis in Section 3.1 shows the top tree-based models stay close and stable across seeds. The wider reproducibility literature says much the same. Across 17 fields, Kapoor and Narayanan [44] found leakage-driven inflation to be the rule rather than the exception, with corrected results frequently no better than plain Logistic Regression. Our own test-set macro-F1 runs from 0.705 to 0.790, and the class sizes are the reason. There are only four Fibrosis, five Hepatitis, and six Cirrhosis cases in the test set. A single wrong prediction therefore swings a per-class F1 hard, and since macro-F1 weights all four classes alike, that swing propagates to the headline number. The same logic reconciles the high AUC-ROC values (0.924–0.982) with the lower macro-F1: AUC rates threshold-independent ranking and mostly overlooks isolated hard-label errors, while macro-F1 absorbs them in full. The gap should therefore be interpreted cautiously; much of it reflects the sensitivity of macro-averaged metrics to extremely small minority-class test counts. Placed alongside prior work, the results are competitive. Butt et al. [10] reported 94.44% validation precision on a proprietary metric applied to a structurally different dataset. Alizargar et al. [9] reported accuracy = 0.95 on the UCI dataset, though under a binary HCV-positive vs. -negative formulation rather than the four-class staging task studied here. Neither work reports cross-validated minority class F1 scores, so direct numerical comparison requires caution.

### 4.2. SHAP Analysis and Clinical Interpretation

Comparing SHAP importance rankings pairwise across all five models reveals the same architecture-dependent structure each time. Tree-based models show strong mutual agreement (rho = 0.923–0.958), pointing to AST, GGT, CHE, and ALP as features that ranked highly across all three regardless of how the trees were built. Such consistency across architectures suggests that AST, GGT, CHE, and ALP appear to carry a robust discriminative signal, which may help highlight which markers warrant closer attention in future externally validated studies. As Figure 13 shows, the three tree-based models (RF, XGBoost, LightGBM) agree strongly with one another (rho = 0.923–0.958) while Logistic Regression and SVM stand apart, agreeing moderately with each other (0.699) and only weakly with the tree models (0.517–0.643). Logistic Regression’s weaker rank agreement does not, however, change the headline feature: it still places AST first, exactly as the other four models do. Alizargar et al. [9] reached the same conclusion through a completely different route, placing AST first and ALP as the second-ranked feature on the UCI HCV dataset using an embedded feature importance method (Figure 4b of their paper)—a finding that corroborates our cross-model SHAP results without any shared methodology. SVM agreed with LR (rho = 0.699) more than with any tree-based model (rho = 0.517–0.566), which may reflect a shared sensitivity to linear feature relationships that both models pick up when inputs are standardised. The stage-specific SHAP gradient tracks the known biochemical course of HCV: at the Donor/Control boundary, normal enzyme levels set the models apart, with AST and GGT as the main distinguishing features; AST and ALP dominate for Hepatitis reflecting active hepatocellular injury [4,5]; AST, ALP, and GGT collectively dominate for Fibrosis, consistent with the combined elevation of hepatocellular injury and cholestatic markers that characterises structural fibrotic disease [4,5]; and CHE rises to the top for Cirrhosis, ranking first in XGBoost and LightGBM and second, behind AST, in Random Forest. CHE’s dominance at the Cirrhosis stage has a clear biological basis: cholinesterase is produced primarily by hepatocytes, so serum levels fall sharply as hepatocyte mass is lost in advanced Cirrhosis [6,7,8]. Clinically, CHE levels below 3506 IU/L predict Cirrhosis with 98.7% sensitivity and 80.3% specificity [6], with strong positive correlation with albumin (r = +0.67, *p* < 0.001) and MELD score [6]—both CHE and albumin decline together as hepatic synthetic function fails. CHE ranked at the top of the Cirrhosis stage in the two boosted tree models and second in Random Forest, and LIME independently placed CHE among the leading Cirrhosis features in the tree-based models, a convergence that strengthens confidence in CHE’s relevance to advanced-stage disease classification.

### 4.3. LIME Analysis and SHAP-LIME Agreement

SHAP and LIME agreed on between one and four of the top five features, depending on the model and disease stage. LR showed the lowest overlap (1/5 for Fibrosis), which fits with the larger gap between LR’s SHAP rankings and those of the tree-based models seen in Section 3.5. Quantitatively, LR achieves the lowest mean overlap across the four disease stages (2.50/5), reflecting its more distinct feature ranking pattern, while Random Forest achieves the highest (4.00/5), reaching 4/5 on every class, unsurprising given that its SHAP rankings align most closely with the overall cross-model pattern. The tree-based models showed consistently moderate-to-high overlap, ranging from 3/5 to 4/5 across all model–class combinations. At the class level, Donor/Control and Hepatitis show the highest mean SHAP-LIME overlap across models (3.6/5 each), because their dominant markers (AST, ALP, and GGT) show up clearly in both global and local explanations. Cirrhosis shows the lowest per-class mean overlap (3.0/5), driven mainly by the SVM 2/5 result, where the local neighbourhood of the representative instance is easily distorted by the surrounding imbalance. Above all, CHE appeared among the top Cirrhosis features in both SHAP and LIME for the tree-based models, two methods that work in completely different ways, which makes this convergence genuinely informative. No other feature–class combination achieved comparable agreement across methods and architectures. Partial disagreement in other model–class combinations, particularly for Logistic Regression, is consistent with SHAP and LIME measuring different aspects of model behaviour [39], one global and the other local. This is also in line with the wider methodological literature: Salih et al. [32] show that the two methods can rank features differently and that correlated inputs distort both, so a degree of divergence is expected rather than a sign of model failure.

### 4.4. DiCE Counterfactual Analysis

These counterfactual outputs are exploratory—they are not treatment recommendations and have not been validated clinically. That said, one can compare the feature changes the model requests against what actually happens biochemically after successful HCV treatment. Several of the feature changes the model calls for are directionally consistent with biochemical improvements reported after successful DAA therapy. Clinical studies have shown that ALT and AST typically normalise within the first two weeks of successful DAA therapy [3]. Cholinesterase recovery follows a similar pattern of hepatic restoration in antiviral treatment settings [6], though data specifically from HCV DAA cohorts are still limited. The four counterfactuals in Figure 20 reach the Donor/Control prediction by different routes: CF3 mainly through a large AST reduction and CF4 through a GGT reduction, while CF1 and CF2 leave the plotted markers unchanged and move only the features not shown (ALT, CHOL, CREA, PROT). This suggests the model recognises several biochemical routes to the healthy state rather than a single fixed boundary. For Patient B, the albumin increase is consistent with improved hepatic synthetic function, since albumin rises when functional hepatocyte mass is restored [6,50]; the accompanying AST and GGT increases, however, should be read as movements in feature space toward the Hepatitis decision boundary rather than as clinically desirable treatment changes, since AST and GGT typically fall rather than rise with hepatic recovery. Patient C’s Fibrosis-to-Donor/Control counterfactual involves an ALP increase (+2.960 SD) and a modest BIL reduction. Because Age and Sex were locked as immutable, none of the counterfactuals proposes a demographic change, so every suggested move falls on a modifiable biochemical marker. These counterfactuals identify decision-boundary changes learned by the model; they should not be read as direct biological recovery pathways or as treatment recommendations, and all outputs still need clinical review before any operational use.

### 4.5. Limitations

Several practical constraints should be kept in mind when reading these results. The UCI HCV dataset is small (615 records) and severely imbalanced, and the macro-F1 standard deviations of 0.031–0.086 across CV folds make clear how much minority class composition varies from fold to fold. Minority classes contain as few as 21 instances (Fibrosis), making it difficult to estimate per-class performance reliably with so little data; the merging of the seven-instance suspect blood-donor group into Donor/Control removed the least reliable class but did not eliminate this constraint. Second, all models used fixed hyperparameters without systematic tuning; while fixing them was intentional—any observed XAI differences between models can then be attributed more directly to architecture rather than tuning—optimising hyperparameters via the CV framework would likely improve minority class recall, especially for SVM. Third, LIME explanation instability under random perturbation sampling was not formally quantified with bootstrap confidence intervals—a known issue in the LIME literature [16]; future work could address it using stability-enhanced variants such as LIME_stability [35].

Fourth, the dataset originates from a single institution (Hannover Medical School, Germany [43]) and the cohort’s composition—in terms of Age distribution, Sex ratio, HCV genotype mix, and comorbidity burden—may not represent patients from other settings, and biomarker importance rankings could shift accordingly [1,21]. A recent umbrella review of HCV across the Eastern Mediterranean Region found pooled prevalence of 28% among healthcare-exposed populations and 31% among key populations such as people who inject drugs [19], distributions that differ markedly from a Western European referral cohort and under which the relative importance of AST, CHE, and ALB could plausibly shift. True external validation on an independent clinical cohort was considered but not performed, because no public HCV dataset shares the UCI feature panel and staging taxonomy: the HCV-Egy dataset [10,51], derived from an Egyptian cohort, uses a histological Fibrosis staging scale (Portal to Cirrhosis) and a feature set of viral-load and longitudinal enzyme measurements that cannot be mapped onto the biochemical Donor/Control–Hepatitis–Fibrosis–Cirrhosis labels used here, so any cross-dataset transfer would confound genuine generalisation failure with feature and label mismatch. As an internal robustness check, the 5-fold evaluation was instead repeated across five random seeds (Section 3.1); macro-F1 remained stable, with XGBoost and LightGBM the closely matched top performers (0.648 ± 0.023 and 0.645 ± 0.026), indicating that the reported rankings are not an artefact of one particular fold assignment, though this check does not substitute for validation on an independent cohort. Multi-centre external validation remains the most important direction for future work.

Fifth, the DiCE counterfactuals are exploratory; a hepatologist should review each output before it is used in any clinical context. Sixth, all preprocessing steps (median imputation, standard scaling, and SMOTE) were fitted independently inside each cross-validation training fold, so no validation-fold information entered model fitting; this fold-level independence is what allows the cross-validated estimates to be read as a fair reflection of generalisation, but it does not remove the underlying scarcity of minority-class data. A residual limitation of the SHAP comparison is that, although all five models are explained on the same fixed 50-instance subset, that subset is a fraction of the full test set; a larger evaluation set would tighten the importance estimates further.

The most useful extensions for future work would include systematic hyperparameter optimisation, external validation on multi-centre datasets, physiologically constrained DiCE with clinician-defined feasible feature ranges, longitudinal biomarker trajectories captured over the course of antiviral treatment, and formal user studies examining clinician trust and adoption. DiCE’s random generation method also does not always honour the permitted_range constraint; in the present run all final counterfactual values fall within the stated ±3.5 bound, but the random generation algorithm can occasionally produce marginal exceptions, a known quirk of the method rather than an error in the model. Since no fixed random seed was applied to the DiCE step, rerunning Figure 20 may produce different counterfactuals, though the core pattern holds across runs: changes in hepatic synthetic or injury-related markers recur across runs, but the specific features vary between counterfactual solutions: ALB moves in several of the representative counterfactuals, while others rely on BIL, AST, GGT, ALP, or unplotted biochemical features. The DiCE outputs should therefore be read as model-space decision-boundary explanations rather than a single consistent biochemical recovery pathway.

## 5. Conclusions

We have described a multi-method explainable AI framework for Hepatitis C stage classification using SHAP, LIME, and DiCE counterfactuals across five ML architectures. The pipeline was built to limit leakage and evaluated with 5-fold stratified cross-validation on the UCI HCV dataset. The cross-validation opened up a wide gap: the best single-split macro-F1 (Logistic Regression, 0.790) fell to 0.598 ± 0.063 once cross-validated, a reminder of how far a single split can mislead on imbalanced clinical data. LightGBM took the best CV macro-F1 in the main run, 0.6840 ± 0.0311. Under repeated-seed cross-validation it and XGBoost remained the two front-runners at almost the same values, 0.645 ± 0.026 and 0.648 ± 0.023, evidence that the leading tree models hold up once the minority classes are re-sampled. To our knowledge, no previous HCV study has used Spearman rank correlation to measure how consistently different ML architectures order the same features by importance. Tree-based models showed strong mutual agreement (rho = 0.923–0.958); that AST, GGT, CHE, and ALP ranked highly across all of them, with AST corroborated by Alizargar et al. [9] through an independent method, suggests these markers carry real discriminative signal. Stage-specific SHAP analysis traced a biochemical progression from healthy to end-stage disease in which CHE emerged as the leading Cirrhosis marker in the two boosted tree models and ranked second behind AST in Random Forest, matching what clinical studies report for CHE as a Cirrhosis predictor (98.7% sensitivity and 80.3% specificity [6]), and independently supported by LIME in the tree-based models. The counterfactual analysis generated what-if scenarios within constrained standardised feature ranges for Cirrhosis and Fibrosis patients, with Age and Sex locked as immutable so that every proposed change fell on a modifiable biochemical marker. The counterfactuals for Cirrhosis patients called for ALB elevation together with changes in BIL, AST, and GGT; the albumin recovery is directionally consistent with improved hepatic synthetic function after successful DAA therapy [3], while the remaining changes are best understood as model decision-boundary movements rather than clinical treatment targets. Priorities for future work include external multi-centre validation, systematic hyperparameter optimisation, physiologically constrained counterfactual generation with clinician-defined feasible ranges, longitudinal biomarker tracking over antiviral treatment, and user studies examining how clinicians engage with these explanations in practice.

## Figures and Tables

**Table 1:** Final hyperparameter values and tuning strategy for all five models.

Model	Key Hyperparameters	Tuning Strategy
Logistic Regression	penalty = l2 (default), C = 1.0 (default), solver = lbfgs, max_iter = 2000	Fixed/default
Random Forest	n_estimators = 100, criterion = gini, max_depth = None (default)	Fixed/default
XGBoost	n_estimators = 100, learning_rate = 0.3 (default), max_depth = 6 (default), eval_metric = mlogloss	Fixed/default
LightGBM	n_estimators = 100, learning_rate = 0.1 (default), num_leaves = 31 (default)	Fixed/default
SVM	kernel = rbf, C = 1.0, gamma = scale (default), probability = True	Fixed/default

Hyperparameters were fixed at library defaults (except max_iter for Logistic Regression and probability = True for SVM) rather than tuned, so that any differences in feature-importance rankings between models can be attributed to architecture rather than to a search over hyperparameters. No grid, random, or Bayesian search was performed.

**Table 2:** Model performance on held-out test set (*n* = 123).

Model	Accuracy	Macro F1	AUC-ROC	Top-5 SHAP Features
Logistic Regression	0.9512	0.7904 *	0.9815	AST, ALP, ALB, CHE, GGT
Random Forest	0.9512	0.7382	0.9825	AST, GGT, CHE, BIL, ALP
XGBoost	0.9431	0.7049	0.9701	AST, CHE, ALP, GGT, BIL
LightGBM	0.9512	0.7632	0.9559	AST, ALP, CHE, GGT, BIL
SVM	0.9431	0.7189	0.9239	AST, GGT, PROT, CHE, ALP

* Best macro-F1 on the held-out test set. AUC = macro one-vs-rest; top-5 SHAP features are computed on the shared 50-instance evaluation subset.

**Table 3:** Five-Fold stratified cross-validation results (mean ± SD). Preprocessing inside each fold.

Model	Accuracy	Macro F1	AUC-ROC
Logistic Regression	0.8829 ± 0.0209	0.5980 ± 0.0630	0.9005 ± 0.0533
Random Forest	0.9398 ± 0.0065	0.6423 ± 0.0401	0.9652 ± 0.0118
XGBoost	0.9350 ± 0.0145	0.6529 ± 0.0615	0.9550 ± 0.0184
LightGBM (Best CV F1)	0.9463 ± 0.0083	0.6840 ± 0.0311 *	0.9593 ± 0.0129
SVM	0.9138 ± 0.0183	0.6224 ± 0.0863	0.9344 ± 0.0340

* LightGBM achieves the best CV macro-F1 (0.6840 ± 0.0311). The Logistic Regression test-split advantage (0.790) does not hold under CV (0.598 ± 0.063), reflecting minority-class instability.

**Table 4:** SHAP-LIME top-5 feature overlap—complete results (all 20 model–class combinations).

Model	Disease Stage	Overlap	SHAP Top-5 Features	LIME Top-5 Features
Logistic Regression	Donor/Control	3/5	AST, ALP, ALB, CHE, GGT	ALP, AST, GGT, BIL, Sex
Logistic Regression	Hepatitis	3/5	AST, ALP, ALB, CHE, GGT	ALP, BIL, Age, CHE, AST
Logistic Regression	Fibrosis	1/5	AST, ALP, ALB, CHE, GGT	CHOL, BIL, AST, Age, PROT
Logistic Regression	Cirrhosis	3/5	AST, ALP, ALB, CHE, GGT	ALB, GGT, PROT, AST, CREA
Random Forest	Donor/Control	4/5	AST, GGT, CHE, BIL, ALP	AST, GGT, BIL, ALP, CHOL
Random Forest	Hepatitis	4/5	AST, GGT, CHE, BIL, ALP	ALP, CHE, BIL, AST, Age
Random Forest	Fibrosis	4/5	AST, GGT, CHE, BIL, ALP	AST, ALB, ALP, CHE, GGT
Random Forest	Cirrhosis	4/5	AST, GGT, CHE, BIL, ALP	ALB, CHE, GGT, ALP, BIL
XGBoost	Donor/Control	4/5	AST, CHE, ALP, GGT, BIL	AST, BIL, GGT, ALP, ALT
XGBoost	Hepatitis	4/5	AST, CHE, ALP, GGT, BIL	ALP, CHE, Age, BIL, AST
XGBoost	Fibrosis	3/5	AST, CHE, ALP, GGT, BIL	AST, ALB, Age, ALP, CHE
XGBoost	Cirrhosis	3/5	AST, CHE, ALP, GGT, BIL	ALB, CHE, BIL, AST, ALT
LightGBM	Donor/Control	4/5	AST, ALP, CHE, GGT, BIL	AST, GGT, BIL, ALP, ALT
LightGBM	Hepatitis	4/5	AST, ALP, CHE, GGT, BIL	ALP, CHE, BIL, Age, GGT
LightGBM	Fibrosis	4/5	AST, ALP, CHE, GGT, BIL	ALP, AST, GGT, ALB, CHE
LightGBM	Cirrhosis	3/5	AST, ALP, CHE, GGT, BIL	ALB, CHE, BIL, ALT, GGT
SVM	Donor/Control	3/5	AST, GGT, PROT, CHE, ALP	AST, ALP, GGT, CHOL, BIL
SVM	Hepatitis	3/5	AST, GGT, PROT, CHE, ALP	ALP, CHE, Age, ALB, AST
SVM	Fibrosis	4/5	AST, GGT, PROT, CHE, ALP	PROT, AST, ALP, GGT, BIL
SVM	Cirrhosis	2/5	AST, GGT, PROT, CHE, ALP	ALT, AST, GGT, ALB, Sex

The lowest overlap is 1/5 (Logistic Regression on Fibrosis) and the highest is 4/5, reached by Random Forest on all four classes, LightGBM on three, and XGBoost on two. CHE appears in the LIME top-5 for Cirrhosis in the three tree-based models, independently confirming the SHAP findings for those models.

**Table 5:** DiCE counterfactual results (standardised units, with permitted_range Constraints of ±3.5 SD; Age and Sex held immutable).

Patient	Target Stage	Feature	Original (SD)	Counterfactual (SD)	Change (SD)
Patient A (Cirrhosis)	Donor/Control	ALB	−1.198	+1.263	+2.461
Patient A (Cirrhosis)	Donor/Control	BIL	−0.045	+2.131	+2.176
Patient B (Cirrhosis)	Hepatitis	ALB	−1.198	+2.418	+3.616
Patient B (Cirrhosis)	Hepatitis	AST	−0.464	+2.242	+2.706
Patient B (Cirrhosis)	Hepatitis	GGT	+1.178	+3.418	+2.240
Patient C (Fibrosis)	Donor/Control	ALP	−1.206	+1.754	+2.960
Patient C (Fibrosis)	Donor/Control	BIL	+0.048	−0.651	−0.699

All values are in standardised units (mean = 0, SD = 1) after preprocessing and scaling. The permitted_range constraint limits the generated counterfactual feature values, not the magnitude of change from the original patient profile; therefore, large change values may occur when the original feature value lies far outside the permitted interval.

**Table 6:** Computational cost (wall-clock runtime) of the XAI methods on the shared 50-instance evaluation subset.

Method	Model	Scope	Runtime (s)
SHAP (TreeExplainer)	Random Forest	50 instances	0.20
SHAP (TreeExplainer)	XGBoost	50 instances	0.10
SHAP (TreeExplainer)	LightGBM	50 instances	0.37
SHAP (KernelExplainer)	Logistic Regression	50 instances	5.96
SHAP (KernelExplainer)	SVM	50 instances	102.49
LIME	LightGBM	1 instance, 5000 samples	0.07
DiCE	LightGBM	1 query, 4 counterfactuals	0.22

Runtimes measured on Google Colab (single CPU session). TreeExplainer computes exact Shapley values and is fastest; KernelExplainer for the non-tree models (Logistic Regression, SVM) is the principal computational bottleneck.

## Data Availability

The UCI HCV dataset used in this study is publicly available from the UCI Machine Learning Repository at https://doi.org/10.24432/C5D612. The full experimental code, including the leakage-controlled preprocessing pipeline, SHAP, LIME, DiCE, and cross-model agreement analyses, is available as a Google Colab notebook at https://github.com/Khalid-S-Alalawi/HCV-MultiXAI-Pipeline (accessed on 23 May 2026).

## Abbreviations

The following abbreviations are used in this manuscript:

ALP	Alkaline phosphatase
ALB	Albumin
ALT	Alanine aminotransferase
AST	Aspartate aminotransferase
AUC	Area under the receiver operating characteristic curve
BIL	Bilirubin
CHE	Cholinesterase
CHOL	Cholesterol
CREA	Creatinine
DAA	Direct-acting antiviral
DiCE	Diverse Counterfactual Explanations
GGT	Gamma-glutamyl transferase
HCC	Hepatocellular carcinoma
HCV	Hepatitis C virus
LGBM	LightGBM (Light Gradient Boosting Machine)
LIME	Local Interpretable Model-agnostic Explanations
LR	Logistic Regression
ML	Machine learning
PROT	Total protein
RF	Random Forest
SHAP	SHapley Additive exPlanations
SMOTE	Synthetic Minority Oversampling Technique
SVM	Support Vector Machine
XAI	Explainable artificial intelligence
XGB	XGBoost (Extreme Gradient Boosting)
